# BRAF Modulates Stretch-Induced Intercellular Gap Formation through Localized Actin Reorganization

**DOI:** 10.3390/ijms22168989

**Published:** 2021-08-20

**Authors:** Anna Hollósi, Katalin Pászty, Miklós Kellermayer, Guillaume Charras, Andrea Varga

**Affiliations:** 1 Department of Biophysics and Radiation Biology, Semmelweis University, H-1094 Budapest, Hungary; hollosi.anna92@gmail.com (A.H.); kcsokay@gmail.com (K.P.); mszkellermayer@gmail.com (M.K.); 2 London Centre for Nanotechnology, University College London, London WC1H 0AH, UK; g.charras@ucl.ac.uk; 3 Department of Cell and Developmental Biology, University College London, London WC1E 6BT, UK

**Keywords:** actin cytoskeleton, BRAF RNAi, endothelial monolayer, intercellular gaps, mechanical stretch

## Abstract

Mechanical forces acting on cell–cell adhesion modulate the barrier function of endothelial cells. The actively remodeled actin cytoskeleton impinges on cell–cell adhesion to counteract external forces. We applied stress on endothelial monolayers by mechanical stretch to uncover the role of BRAF in the stress-induced response. Control cells responded to external forces by organizing and stabilizing actin cables in the stretched cell junctions. This was accompanied by an increase in intercellular gap formation, which was prevented in BRAF knockdown monolayers. In the absence of BRAF, there was excess stress fiber formation due to the enhanced reorganization of actin fibers. Our findings suggest that stretch-induced intercellular gap formation, leading to a decrease in barrier function of blood vessels, can be reverted by BRAF RNAi. This is important when the endothelium experiences changes in external stresses caused by high blood pressure, leading to edema, or by immune or cancer cells in inflammation or metastasis.

## 1. Introduction

**Figure 1:**
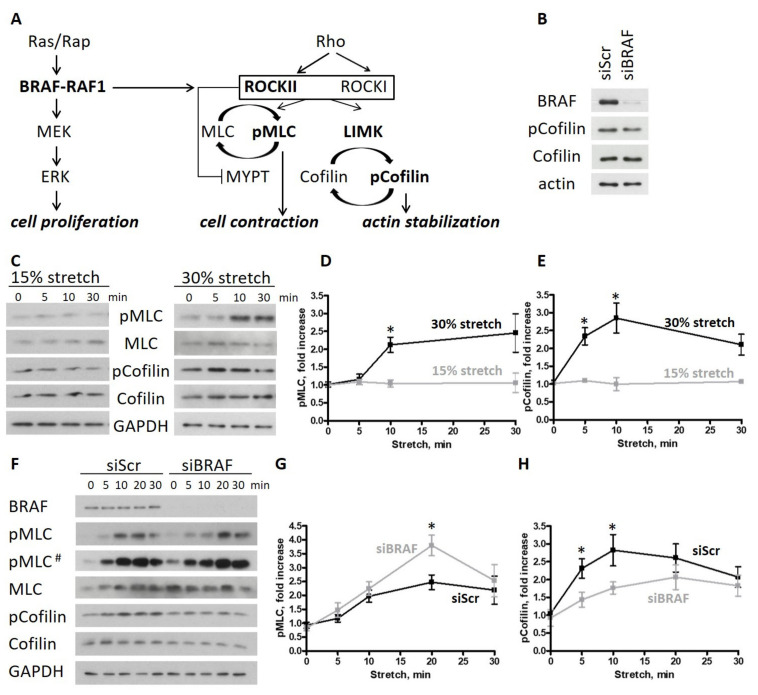
BRAF knockdown increases cell contractility and actin turnover upon stretch in endothelial monolayers. (**A**) Cross-talk of the RAF and ROCK signaling pathways: in mouse endothelial cells, BRAF is necessary to create the RAF1 phosphospecies competent for ROCKII binding. The presence of Rap and RAF1 is essential for the localized ROCKII activity at cell–cell junctions. (**B**) Cofilin phosphorylation of scrambled and BRAF siRNA-transfected HUVECs was analyzed by Western blotting. (**C**) HUVEC monolayers stretched either by 15% or 30% were harvested at different time points after stretch. Phosphorylation of MLC and cofilin was analyzed by Western blotting. Quantification of the data is shown in (**D**) for pMLC and in (**E**) for p-cofilin. (**F**) Scrambled or BRAF siRNA-transfected HUVEC monolayers stretched by 30% were harvested at different time points. Phosphorylation of MLC and cofilin was analyzed by Western blotting. Quantification of the data is shown in (**G**) for pMLC and in (**H**) for p-cofilin. Black line represents the scrambled HUVECs, gray line the BRAF siRNA-transfected HUVECs. The amounts of pMLC and p-cofilin were normalized to the total MLC or cofilin amounts, respectively. Mean ± SEM is plotted. The results shown were from 3–7 independent experiments, carried out by using 3 independent LOTs of HUVECs; # represents a longer exposure; * denotes *p* < 0.05.

The endothelium serves as a barrier between the blood and the surrounding tissues. The proper dynamics of the interconnected endothelial cells are essential for inflammation and cancer metastasis formation, when immune and cancer cells make their way through the endothelial monolayer to reach the underlying tissue [1,2]. We have previously found that the MEK/ERK kinase BRAF modulates these processes, as the endothelial barrier function was strengthened in BRAF knockout mouse endothelial cells (BKO ECs) [3]. The decreased vascular permeability in BKO ECs correlated with less metastasis formation in BKO mice. The underlying mechanism is linked to a perturbation in actin cytoskeleton dynamics. Normally, treatment with permeability-increasing agents induces a shift from linear to focal adherens junction formation in endothelial cells [4]. Linear adherens junctions are composed of cortical actin networks and bundles that run in parallel with cell–cell junctions and feature a so-called circumferential actin ring. Myosin II stabilizes these actin structures and supports cell–cell adhesion stability [5]. Permeability-increasing agents that activate RhoA induce radial stress fiber formation in the center of the cell and increase central cell contractility with myosin II. This process leads to intercellular gap formation and is characterized by punctate VE-cadherin (actin-rich) structures bridging the adjacent cells at cell–cell junctions. Gap formation is required for the paracellular migration of immune or cancer cells. The MEK/ERK kinase RAF1 is responsible for the localization of the cytoskeleton-regulating kinase ROCKII (also known as ROKα) at cell–cell junctions in mouse ECs [6]. The presence of the other RAF isoform BRAF is required for RAF1 to fulfill this function (Figure 1A). Interestingly, in the absence of BRAF, more ROCKII is delivered to the VE-cadherin junctions by RAF1, which strengthens the circumferential actin ring, stabilizes cell–cell contacts and decreases vascular permeability and metastasis formation. This phenotype cannot be reverted by a kinase-dead BRAF mutant, indicating that BRAF kinase activity is necessary to induce the specific RAF1 phosphospecies for the proper RAF1–ROCKII interaction and ROCKII activity at cell–cell junctions [7]. ROCK proteins fulfill several functions in the cell by acting on various substrates [8]. One of these functions is to induce stress generation in F-actin networks by myosin (Figure 1A). This function is realized in several (parallel) ways and results in increased myosin light chain (MLC) phosphorylation on residues Ser19 and/or Thr18 that finally increases myosin ATPase activity and enhances contraction [9]. ROCK can directly phosphorylate MLC, as well as inhibit the function of the MLC phosphatase MYPT1. Another function of ROCKs is to limit actin depolymerization and severing by cofilin (Figure 1A). ROCK phosphorylates LIMK, which in turn phosphorylates cofilin on residue Ser3 and inactivates it. Thus, this dual role of ROCK can affect both actin reorganization and its contraction, both of which are major determinants of intercellular gap formation. Indeed, overexpression of LIMK1 was shown to increase the number of intercellular gaps, while its depletion resulted in enhanced transendothelial electrical resistance (TER), implying an improved barrier function [10]. The other LIMK isoform LIMK2 was shown to phosphorylate cofilin and induce stress fiber formation [11].

Application of mechanical force can also induce the reorganization of adherens junctions [12]. Cadherin-mediated cell–cell adhesions are essential for the transmission of external forces. Cadherins are connected to the actin cytoskeleton by a multiprotein complex composed of p120 catenin and β- and α-catenins. The latter contains a cryptic binding site for vinculin, which is exposed upon force application, binds F-actin and thus induces the reorganization of cortical actin filaments. This is one way to reinforce cell–cell adhesion by promoting cadherin clustering and stabilization [13]. In response to tension, the RhoA pathway becomes activated. In cells, stretching actin filaments increases their affinity to the myosin II motor protein, but not to myosin I [14]. This will not only enhance filament contraction (by ROCK-mediated MLC phosphorylation), but cross-linking of actin cables by myosin II will stabilize the newly formed actin network [15]. In addition, in vitro force application on single actin filaments showed that an increase in fiber tension reduces the binding of cofilin, preventing fiber disassembly [16]. Thus, cells respond to external forces by actin filament stabilization and stress fiber assembly.

Since BRAF is crucial in modulating the dynamics of the actin cytoskeleton, our study aims to elucidate the molecular and cellular mechanism of BRAF’s contribution to the force-induced response of human endothelial cells. To uncover the role of BRAF in endothelial mechanotransduction, we knocked down BRAF expression in endothelial cells, then stretched endothelial monolayers and compared the responses of control and BRAF knockdown cells in two ways: (1) at the molecular level, we analyzed the phosphorylation status (activation/inactivation) of the actin-regulating proteins MLC and cofilin and (2) at the cellular level, we imaged F-actin reorganization in endothelial monolayers in response to stretch and compared the localization changes of pMLC and cofilin of the unstretched and stretched cells. Our results show that there is a more intense actin reorganization upon BRAF knockdown with the formation of stress fibers spanning the whole cell. These fibers might contribute to the strengthening of the junctions, thereby providing a mechanism of how endothelial cells in the absence of BRAF prevent intercellular gap formation upon stretch.

## 2. Results

### 2.1. BRAF Knockdown Increases Cell Contractility and Actin Turnover upon Stretch in Endothelial Monolayers

We previously observed that BRAF KO mouse endothelial cells (ECs) show significant circumferential actin ring formation [3]. This phenotype was associated with decreased cofilin phosphorylation and was connected to the increased binding and inhibition of the Rho-kinase ROCKII by RAF1 in the absence of BRAF. Thus, BRAF has a prominent effect on actin cytoskeleton dynamics in mouse ECs.

Interestingly, in human umbilical vein endothelial cells (HUVECs) cultured in standard conditions, cofilin phosphorylation was not decreased upon BRAF knockdown (Figure 1B). Since the endothelium is exposed to mechanical stimuli in vivo, we decided to examine the role of BRAF in a physiologically more relevant situation and challenged HUVEC monolayers by subjecting them to mechanical deformation. Physiological stretch results in a 5–10% elongation, while pathological conditions can induce elongations of more than 20% in human pulmonary artery endothelial cells and HUVECs [17,18,19]. In our studies, we applied stretch up to 30% to analyze the molecular and cellular responses of HUVECs. First, we determined the extent of stretch necessary to reorganize the actin cytoskeleton by monitoring the phosphorylation status of two regulatory proteins, MLC and cofilin, by Western blotting. We applied uniaxial stretch on HUVEC monolayers with a magnitude of 15% or 30% strain for different durations. We found that both MLC and cofilin phosphorylation increased at 30% stretch after 10 min (Figure 1C and quantifications in Figure 1D,E). By contrast, 15% stretch failed to induce a response.

To determine how BRAF affects the stretch-induced response of HUVECs, we knocked down BRAF with siRNA. The control (siScr) and siBRAF-transfected monolayers were stretched by 30%, and the phosphorylation of MLC and cofilin was monitored by Western blotting (Figure 1F and quantification in Figure 1G,H). MLC phosphorylation was increased in both cases with a peak in phosphorylation at 20 min after stretch. Interestingly, siBRAF-transfected monolayers showed a remarkable, significant increase in pMLC compared to the control cells at the peak value. Cofilin phosphorylation was also increased upon stretch in both cases. However, upon BRAF knockdown, it was phosphorylated to a lesser extent than in siScr-transfected cells.

Taken together, stretch increases myosin activity and switches off cofilin by phosphorylating it. Comparison of the stretch-induced response of control and BRAF knockdown monolayers suggests that, in the presence of BRAF, cofilin is inactivated faster which may lead to an earlier inhibition of actin depolymerization and stabilization of actin cables. It is known that LIMK, the upstream kinase responsible for cofilin phosphorylation, is required for increased stress fiber formation [11]. Therefore, the observed faster cofilin phosphorylation in the presence of BRAF might be translated at the cellular scale as an earlier assembly of stress fibers. In the absence of BRAF, the slower cofilin phosphorylation may allow more actin reorganization and delayed actin stabilization.

### 2.2. ROCKII Is Required for the Phosphorylation of Cofilin upon Stretch in Endothelial Monolayers

**Figure 2:**
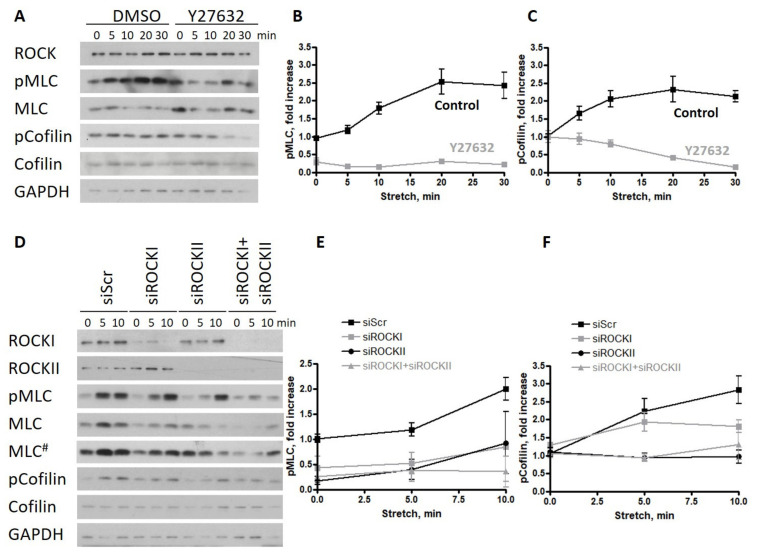
ROCKII is required for the phosphorylation of cofilin upon stretch in endothelial monolayers. (**A**) Effect of the ROCK kinase inhibitor Y27632 was analyzed on the phosphorylation of MLC and cofilin of HUVECs stretched by 30% and harvested at different time points after stretch. Quantification of the data are shown in (**B**) for pMLC and in (**C**) for p-cofilin. (**D**) Scrambled, isoform-specific ROCKI and ROCKII siRNA as well as both in combination has been used to analyze the isoform-specific effect of ROCK on HUVECs stretched by 30% and harvested at different time points after stretch. Quantification of the data is shown in (**E**) for pMLC and in (**F**) for p-cofilin. The amounts of pMLC and p-cofilin were normalized to the total MLC or cofilin amounts, respectively. Mean ± SEM is plotted. For Y27632 treatment, the quantified data are from 2 independent experiments. The results shown for the ROCK siRNA treatment are from 3 independent experiments; # represents a longer exposure.

ROCK activation by stretch is a general property of epithelia/endothelia [20], but it is still an open question as to which ROCK isoform is responsible for the stretch-induced phosphorylation of MLC and cofilin in human cells, and whether the observed decrease in cofilin phosphorylation upon BRAF KD can be phenocopied by ROCK isoform-specific KD. This is particularly interesting since, in mouse ECs, the knockout of RAF1 resulted in the malfunction of the ROCKII (or ROKα) isoform at the adherent junctions [6]. To answer this question, first, we used the inhibitor Y27632, which is a selective inhibitor of Rho-kinases, although it is not isoform specific, to show the role of ROCK kinases in the biological response of HUVECs to stretch. Consistent with previous studies, Y27632 decreased the phosphorylation of MLC significantly and diminished the phosphorylation of cofilin (Figure 2A and quantifications in Figure 2B,C). To determine the relative importance of ROCK isoforms in the cytoskeletal response, we silenced either ROCKI or ROCKII or both kinases. We found that the reduced expression of either isoform decreased the phosphorylation of MLC (Figure 2D and quantification in Figure 2E) but did not prevent its stretch-induced increase (there was no significant difference between ROCKI or ROCKII knockdown and control samples 10 min after stretch). When both isoforms were silenced, the stretch response of the monolayer was completely prevented. Stretch-induced cofilin phosphorylation was preferably affected by ROCKII knockdown, and this decrease was comparable to the double knockdown of both ROCKI and ROCKII (Figure 2D and quantification in Figure 2F). There was no significant difference between ROCKI knockdown and control samples.

These data suggest that ROCKII is required for the stretch-induced cofilin phosphorylation of HUVECs.

### 2.3. The Presence of BRAF Accelerates the Stabilization of the Newly Formed Actin Stress Fibers after Stretch in the Stretched (Horizontal) Junctions

**Figure 3:**
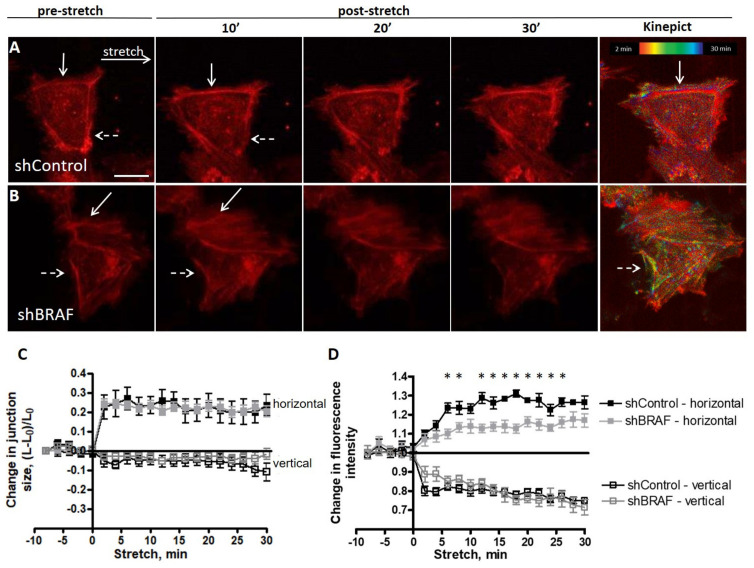
The presence of BRAF accelerates the stabilization of the newly formed actin stress fibers after stretch in the stretched (horizontal) junctions. (**A**,**B**) Example of actin localization changes upon 30% stretch visualized by expressing LifeAct-mCherry in shControl (**A**) and shBRAF (**B**) cells. Pictures before and after stretch at the indicated time points are shown. Scale bar is 25 µm. Solid arrows indicate fluorescence intensity increase in horizontal junctions, while dashed arrows show a decrease in fluorescence intensity in the vertical junctions. The last panel shows the effective half-time calculated by the Kinepict software from the actin fluorescence intensities during the time course of stretch, indicating the time of stretch when the actin fluorescence intensity reached half of the total fluorescence intensity change. The color code is shown in the last figure. For the zoomed-out view of the monolayers, see Figure S1B,C and for the time-lapse images, Videos S1 and S2. (**C**) The extent of stretch in horizontal (filled squares) and vertical (open squares) junctions was calculated using the formula (L − L_0_)/L_0_, where L_0_ is the length of the junction measured before stretch, L stands for the length of the junction measured at the indicated time point after stretch. Data of shControl cells are represented by black, while data of shBRAF cells are in gray. (**D**) Quantification of actin fluorescence intensity changes at the indicated time points after stretch in the horizontal (filled squares) and vertical (open squares) junctions of shControl- (black) and shBRAF-transfected (gray) cells is shown. Data for individual cell junctions can be seen in Figure S1D–G. Three to five cells were analyzed from at least three independent experiments used by two different shRNAs for shBRAF. Mean ± SEM is plotted for each quantification; * denotes *p* < 0.05.

To gain mechanistic insight into how F-actin reorganization is affected by BRAF, we co-expressed shBRAF and the fluorescently labeled actin-binding peptide LifeAct-mCherry in HUVECs. The efficiency of BRAF depletion in the shBRAF clones was verified by Western blotting (Figure S1A). The stretch-induced actin rearrangement was followed by time-lapse confocal microscopy. Confluency of the monolayers was verified by the plasma membrane stain DeepRed CellMask. BRAF knockdown cells were identified by their EGFP expression (EGFP and shBRAF are expressed from the same plasmid under the same promoter). Examples of the analyzed monolayers are shown in Figure S1B,C and zoomed-in views are presented in Figure 3A,B.

To determine how stretch influences junctions, we quantified separately the horizontal (parallel to the direction of stretch) and vertical (perpendicular to the direction of stretch) junction size before and after stretch (Figure 3C). The extent of strain in each junction was calculated as the actual length (L) minus the original length (before stretch) (L_0_), divided by the original (unstretched) length (L_0_) of the analyzed junction [13]. The vertical junction size was not changed upon stretch, while the horizontal junction size was increased by 20–30%, consistent with the imposed stretch.

To gain mechanistic insight into how actin reorganization is affected by BRAF knockdown upon stretch, we have analyzed the stretch-induced relocalization of actin in control and BRAF knockdown monolayers. Within a few minutes of stretch, actin filaments were accumulated in the stretched (horizontal) junctions (Figure 3A,B, zoomed-out view in Figure S1B,C and Videos S1 and S2) and were depleted in the unstretched (vertical) junctions. Quantification of the mean fluorescence intensities showed a 20–30% increase in actin fluorescence intensity both in the presence and in the absence of BRAF in the stretched junctions (arrows in Figure 3A,B, quantification in Figure 3D), and a concomitant 20–30% decrease in the unstretched junctions (dashed arrows in Figure 3A,B, quantification in Figure 3D). Interestingly, control cells reorganized their actin cytoskeleton faster and the actin cables were stabilized 10 min after stretch, while this process in shBRAF cells is slower and it approaches the plateau of the control cells only by 30 min of stretch (quantification of actin fluorescence intensities in individual horizontal cell junctions of shControl and shBRAF cells is shown in Figure S1D,E, while the ones in vertical junctions are illustrated in Figure S1F,G). As we demonstrated earlier (Figure 1F,H), at the molecular level, cofilin phosphorylation kinetics are delayed in shBRAF cells compared to control cells. This may suggest that actin reorganization takes longer in shBRAF cells (30 min after stretch) compared to control cells (10 min after stretch).

In order to visualize the difference in actin reorganization kinetics, we used the Kinepict software (https://kinepict.com/kmit.xhtml#kmit, date accessed: 20 May 2020) to determine the time when the stretch-induced increase in fluorescence intensity in each pixel reached half of the maximum change in that pixel, called the effective half-time. The algorithm is independent of whether an increase or decrease occurred. Effective half-times are then displayed as color-coded images with pixel colors ranging from red (faster changes) to blue (slower changes) (Figure 3A,B). The algorithm, however, also recognizes cell movements as a fluorescence intensity increase or decrease due to pixel shift during the movement. We compared shControl and shBRAF cells, taking the first time point after stretch as the initial one. The last panels in Figure 3A,B illustrate the color-coded images of the corresponding experiments shown in Figure 3A,B and the time series of Videos S1 and S2 analyzed from 2 to 30 min after stretch. Interestingly, in control cells, the actin fluorescence intensity changes are mostly restricted to the cell borders (shown by an arrow in Figure 3A). In shBRAF cells, actin fluorescence intensity changes can be visualized in the whole cell, not only at the junctions, and reorganizations happen later in the vertical junction (dashed arrow in Figure 3B) compared to control cells. This is in line with the quantification of actin fluorescence intensities shown in Figure 3D. The slower kinetics of cofilin phosphorylation observed at a molecular level (Figure 1F,H) may indicate a more intense reorganization of actin in the absence of BRAF but these actin cables appear to be stabilized later compared to the control cells.

Taken together, at the cellular level, the presence of BRAF appears to accelerate the reorganization and recruitment of F-actin to horizontal junctions. In BRAF-depleted monolayers, the actin reorganization is not restricted to the junctions, but also occurs in the whole cell and the actin cables are stabilized later.

### 2.4. The Stretch-Induced Response of the Actin Cytoskeleton Results in a More Efficient Reinforcement of Cell–Cell Junctions through Excess Stress Fiber Formation in the Absence of BRAF

**Figure 4:**
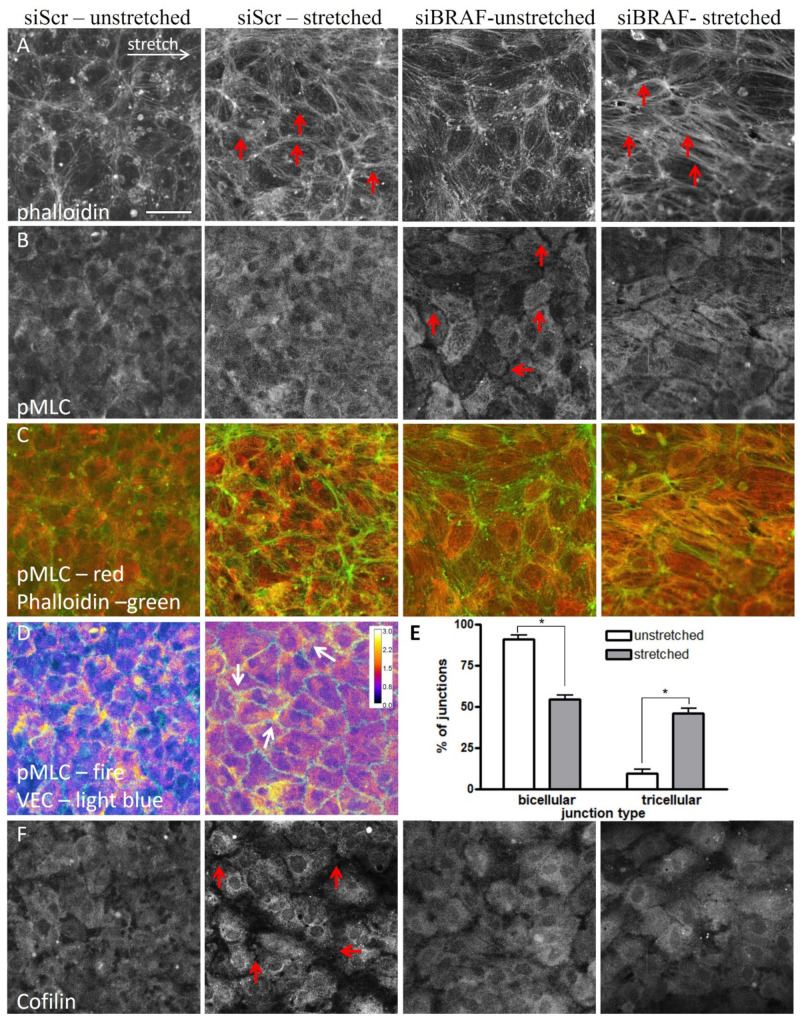
The stretch-induced response of the actin cytoskeleton results in a more efficient reinforcement of cell–cell junctions through excess stress fiber formation in the absence of BRAF. Immunofluorescence images of unstretched and stretched siScr and siBRAF monolayers stained for actin (**A**), pMLC (**B**) and cofilin (**F**). Panel (**C**) shows the merged pMLC (red) and actin (green) stainings. See also Figure S2A for p-cofilin as well as Figure S2B for the merged VE-cadherin and actin staining. Red arrows indicate thicker actin fibers (panel A) in the stretched control and siBRAF cells, and faint pMLC staining at the cell periphery in siBRAF cells (panel B). Scale bar is 50 µm. (**D**) pMLC staining of unstretched and stretched siScr monolayers colored as fire in ImageJ to illustrate the regions with increased pMLC staining (yellow). VE-cadherin staining is shown in light blue. Arrows indicate higher pMLC staining in the tricellular junctions of the stretched monolayer. (**E**) Quantification of bi- and tricellular junctions plotted as % of junctions in unstretched (white bars) vs. stretched (gray bars) conditions. Mean ± SEM is plotted, data are from 90–100 junctions. The results are from 2–3 independent experiments; * denotes *p* < 0.05.

To determine how the difference in MLC phosphorylation between control and shBRAF monolayers (Figure 1F,G) affects actin stress fiber formation and contraction, we fixed control and siBRAF monolayers and stained them with phalloidin (Figure 4A) and for pMLC (Figure 4B) both in the unstretched condition and 15 min after stretch. Figure 4C illustrates the composite image of pMLC and F-actin (phalloidin) stainings. Comparison of phalloidin staining of unstretched control (siScr) and siBRAF cells shows that siBRAF cells have more peripheral actin. This is in agreement with the staining pattern of BRAF KO mouse endothelial cells [3]. Upon stretch, as expected, in control cells parallel thin actin cables appear in the direction of stretch, and, moreover, thicker ones are formed in the horizontal junctions. Upon BRAF knockdown, the actin cables are thicker not only in the horizontal junctions but also within the cells (see red arrows in Figure 4A). Interestingly, unstretched control and siBRAF monolayers show a completely different staining pattern for pMLC (Figure 4B). In control cells, the distribution of pMLC is homogenous while siBRAF cells show very faint staining close to the cell periphery (i.e., at cell–cell junctions, see red arrows) and pMLC seems to be structured in other parts of all cells. Upon stretch, pMLC staining becomes more pronounced at control cell vertices, where more than two cells meet, i.e., in tricellular junctions (Figure 4D and quantification in Figure 4E). In siBRAF cells, pMLC becomes more structured and colocalizes with actin stress fibers not only in the horizontal junctions, but also inside the cells (Figure 4A–C). At the cellular level, the observed enhanced colocalization of actin stress fibers with pMLC may indicate an increased contractility and, indeed, we obtained increased MLC phosphorylation at the molecular level in BRAF-depleted cells upon stretch (Figure 1F,G).

To explore further how the observed cofilin phosphorylation (Figure 1F,H) contributes to the different actin stabilization kinetics, we stained the unstretched and stretched monolayers for both cofilin (Figure 4F) and p-cofilin (Figure S2A). As expected, in unstretched control cells, cofilin staining was homogenous [16]. Strikingly, it became more perinuclear upon stretch, and the fluorescent signal decreased significantly at cell–cell junctions. p-cofilin staining was uniform within the cells and did not change upon stretch. This points at the presence of less active cofilin at the junctions in stretched cells. This is in line with the observed stabilization of actin cables in the stretched junctions, from which the non-phosphorylated, active cofilin is excluded. In siBRAF cells, both cofilin and p-cofilin stainings showed a uniform distribution and none of them changed upon stretch. This is in line with our finding that in the absence of BRAF, actin reorganization happens within the whole cell and it is not restricted to the junctions (Figure 3A,B).

**Figure 5:**
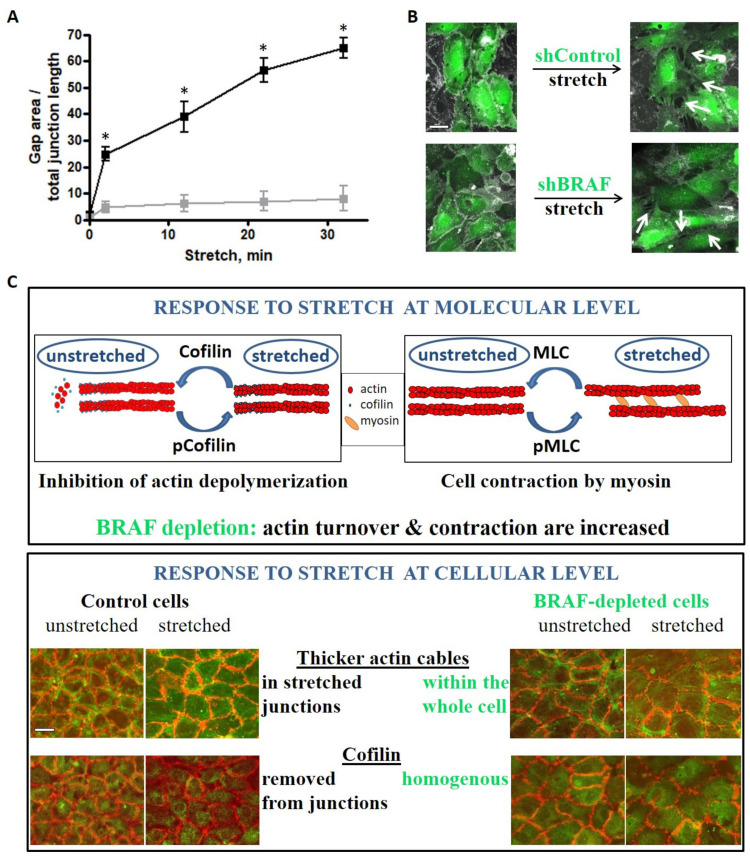
Stretch-induced gap formation is prevented between BRAF-depleted endothelial cells. (**A**) Quantified changes in intercellular gap formation in shControl (black) and shBRAF (gray) monolayers. The results shown are from 4–5 independent experiments, * denotes *p* < 0.05. Panel (**B**) shows an example of shControl and shBRAF RNA-expressing monolayer before and 30 min after stretch. Videos S3 and S4 show the time-lapse images of the monolayers shown in (**B**). Plasma membrane stain (DeepRed CellMask) is colored as gray, cells expressing shControl or shBRAF RNA are labeled in green. Scale bar is 25 µm. (**C**) Summary of the stretch-induced response of endothelial cells at molecular and cellular levels and the effect of BRAF depletion. At the molecular level, both cofilin and MLC are phosphorylated upon stretch. Cofilin is inactivated by phosphorylation, therefore actin depolymerization/turnover is inhibited. MLC phosphorylation accelerates actin contraction by myosin. Upon BRAF knockdown, actin turnover is increased (cofilin phosphorylation/inactivation is delayed) and actin contraction is also increased. At the cellular level, control cells accumulate actin fibers in the horizontal (stretched) junctions, from which cofilin is excluded. This might be interpreted as actin fibers being stabilized in the stretched junctions. Upon BRAF knockdown, thicker actin fibers are found throughout the whole cell and not restricted to the stretched junctions only (from where cofilin is not excluded). Scale bar is 50 µm.

We analyzed intercellular gap formation upon stretch as an indicator of cell–cell junction stability in live cells (Figure 5A,B and Videos S3 and S4). Gap formation in control monolayers increased approximately 15-fold right after stretch and increased further over time. Interestingly, shBRAF monolayers already displayed slightly fewer gaps (about half of the control) before stretch. This observation can be explained by the increased peripheral actin ring formation of the BRAF-depleted cells (Figure S2B). Intriguingly, the gap area increased about 5-fold right after stretch and did not increase much further over time in the absence of BRAF. Presumably, the increased contractility of BRAF knockdown cells makes them capable of adjusting their shape faster upon stretch and therefore they can strengthen their junctions and minimize gap formation.

Taken together, BRAF is important for limiting stress fiber formation and for the localized activity of cofilin upon stretch. Interestingly, the observed increase in stress fiber formation in BRAF knockdown cells does not enhance intercellular gap formation, rather it contributes to the faster adaptation of cell shape to the external stress, which finally leads to more efficient reinforcement of cell–cell junctions.

## 3. Discussion

The dynamic nature of the intercellular adhesion of endothelial cells is critical for barrier integrity, determines the extent of inflammation and hence affects the efficiency of tumor metastasis formation [21]. The stability of cell–cell adhesion is determined by a balance between intrinsic forces generated by the actomyosin machinery of the endothelial cells and extrinsic forces [13,22], such as shear stress caused by blood flow, blood pressure (tensile stress), vessel wall contraction and forces exerted by penetrating immune or cancer cells [4,17]. Shear stress activates the Rho-ROCK-LIMK-cofilin pathway [23], which in turn regulates the actin cytoskeletal response. The pulsatile nature of the blood flow causes a permanent exposure of endothelial cells to cyclic mechanical strain. Actin filaments are organized perpendicularly to the direction of the applied cyclic stress, allowing cells to maintain their structure with minimal changes in intracellular tension [18]. This reorganization is accompanied by increased MLC phosphorylation [24]. Cyclic stretch-induced MLC phosphorylation can be completely abolished by the application of the ROCK inhibitor Y27632, thus, endothelial cells reorganize their actin cytoskeleton upon cyclic stretch by ROCK-mediated MLC phosphorylation.

In our experiments, we also observed enhanced MLC phosphorylation in control endothelial monolayers upon unidirectional stretch. Endothelial cells react to the applied external stress not only with increasing contraction, but they also control their actin turnover through ROCK kinases impinging on cofilin phosphorylation. ROCKII has a greater contribution to the stretch-induced cofilin phosphorylation than ROCKI, providing another example of ROCK isoform-selective substrate specificity [25,26]. The amount of active cofilin determines the rate of actin depolymerization and is responsible for removing the original actin cables formed under low tension [27,28]. In the presence of BRAF, cells react to the external stress by localizing their actin filaments in the stretched (horizontal) junctions, a phenomenon which was also observed in epithelial cells [27,29]. Cofilin is excluded from those junctions by the time F-actin becomes stabilized there (Figure 5C). Upon external stress, there is an increase in intercellular gap formation, which might be due to the weakening of cell–cell junctions, mostly in higher-order junctions, where more than two cells meet. In these so-called tricellular junctions, vertices experience high tension, since the line tension from the connected cell edges accumulates at the vertices [30]. Indeed, vertices were the structures where the fracture of epithelial monolayers was initiated upon the induction of cell contractility by calyculin A treatment [31]. Vertices were also identified recently as sites where intercellular gaps are spontaneously formed in the endothelial monolayer and where cancer cells preferentially extravasate [32].

In the absence of BRAF, the molecular scale response is realized in more contractility and slower cofilin phosphorylation, which at a cellular scale is translated into more intense stress fiber formation, with fibers spanning the whole cell (Figure 5C). In addition, active cofilin fails to be removed from the junctions upon stretch, which may correlate with the fact that actin reorganization and stress fiber formation are not restricted to the junctions. However, in the absence of BRAF, external stress-induced gap formation is prevented. BRAF knockdown cells might reinforce their cell–cell junctions through increased stress fiber formation. In fact, these cells are more contractile and there is a more intense reorganization that ultimately results in faster adaptation of cell shape to the external stress and an increased barrier function of BRAF-deficient monolayers. Thus, BRAF, by influencing the local activity of ROCKII, modulates the barrier function of endothelial cells impinging on VE-cadherin-based adherent junctions. Our study raises the question whether BRAF exerts this function in other cellular contexts, e.g., by modulating the immune response downstream of the adhesion molecule ICAM-1.

The barrier function of the endothelium needs to be tightly regulated to allow a controlled gap opening–closing for a proper immune response towards pathogens [33]. However, chronic inflammation is associated with sustained gap formation in many diseases, e.g., asthma, ischemic stroke and cancer. In such cases, downregulation of BRAF might help to strengthen cell–cell junctions. There are already approaches for optimized siRNA delivery to specific organs or cell types such as lung endothelial cells [34,35] that may be applicable in humans to treat different diseases in the near future.

Stretching of endothelial cells can happen in a minute timescale during exercise-induced vasodilation [36]. Our results suggest that external stress, through an increased gap formation, can induce a permeability increase in the endothelial barrier. Indeed, an increase in permeability [37,38] or increased inflammation [39] was observed upon the application of mechanical stresses. Since BRAF ablation in mice decreases endothelial permeability [3], downregulation of BRAF might also help to reduce external stress-induced permeability increase in human cells, to reduce inflammation. In addition, endothelial cells can be physiologically stretched as a consequence of elevated blood pressure, especially in grafted veins experiencing higher (arterial) pressures when grafted into arteries [40,41]. Venous hypertension results in inflammation and vascular wall remodeling and then increased incidence of edema in the capillaries [42].

## 4. Materials and Methods

### 4.1. Reagents

Primary antibodies for Western blotting (WB): actin (I-19), Cat# sc-1616, Santa Cruz Biotechnology; BRAF, Cat# 14814S; cofilin (both WB and IF), Cat# 5175S; GAPDH, Cat# 97166S; MLC, Cat# 8505S; p-cofilin (both WB and IF), Cat# 3313S; pMLC (Thr18/Ser19), Cat# 3674S; all from Cell Signaling; ROCKI, Cat# 611136; ROCKII, Cat# 610623; both from BD Biosciences.

Primary antibodies for immunofluorescence (IF): VE-cadherin, Cat# 2500S; PECAM-1, Cat# 3528S; pMLC (Ser19), Cat# 3675S; all from Cell Signaling.

Secondary antibodies for WB: Peroxidase AffiniPure Goat Anti-Mouse IgG (H + L), Cat# 115-035-003; Peroxidase AffiniPure Goat Anti-Rabbit IgG (H + L), Cat# 111-035-003; Peroxidase AffiniPure Donkey Anti-Goat IgG (H + L), Cat# 705-035-003; all from Jackson ImmunoResearch.

Secondary antibodies for IF: Chicken Anti-Rabbit IgG (H + L) Cross-Adsorbed Secondary Antibody, Alexa Fluor 488, Cat# A21441; Goat Anti-Mouse IgG (H + L) Cross-Adsorbed Secondary Antibody, Alexa Fluor 546, Cat# A11003; both from ThermoFisher Scientific; Phalloidin CruzFluor™ 647 Conjugate, Cat# sc-363797 from Santa Cruz Biotechnology.

siRNAs: siScr: ON-TARGETplus Non-targeting Control Pool, Cat# D001810-10-20; siBRAF: ON-TARGETplus Smart Pool Human BRAF, Cat# L-003460-00-0020; both from Dharmacon. siROCKI, Cat# NM_005406, SASI_Hs01_00065570; siROCKII, Cat# NM_004850, SASI_Hs01_00204251; both from Sigma.

Bacterial strains: TOP10 chemically competent *E. coli*, Cat# C404010; Stbl3 chemically competent *E. Coli*, Cat# C737303 were from ThermoFisher Scientific.

Chemicals for cell culture: basic Fibroblast Growth Factor, Cat# F0291; gelatin, type B, 2 % solution, Cat# G1393; heparin, Cat# H3149; Hydrocortison, Cat# H0396; polydopamine, Cat# H8502; vitamin C, Cat# A4544; all from Sigma. Chemically Defined Lipid Concentrate, Cat# 11905031; DMEM, Cat# 10313021; EGF, Cat# PHG0311; GlutaMAX Supplement, Cat# 35050038; HBSS, Cat# 14025050; HEPES, 1M Buffer Solution, Cat# 15630049; insulin–transferrin–selenium, Cat# 41400045; L-Glutamine, Cat# 25030081; MCDB-131 medium, Cat# 10372019; penicillin–streptomycin, Cat# 15140148; all from ThermoFisher Scientific. fetal bovine serum, Cat# P40-39500 from PAN Biotech. ROCK inhibitor, Y27632, Cat# 13624S from Cell Signaling.

### 4.2. Cultured Cells/Cell Lines

HUVEC cell culture. HUVECs were purchased from Caltag Medsystems (UK) (Cat# ZHC-2301) and were cultured in MCDB medium, supplemented with 5% fetal bovine serum, 1% penicillin–streptomycin, 1% Chemically Defined Lipid Concentrate, 1% HEPES, 1% GlutaMAX Supplement, 0.3% insulin–transferrin–selenium, 1 ng/mL basic Fibroblast Growth Factor, 2 ng/mL EGF, 5 µg/mL vitamin C and 250 nM hydrocortisone. Tissue culture dishes were coated with 0.5% gelatin for proper attachment of HUVECs. All cell culture was performed at 37 °C in a humidified atmosphere containing 5% CO_2_. Monolayer stretching was carried out in HBSS, supplemented with 5% fetal bovine serum, 1% HEPES and 1% penicillin–streptomycin.

HEK293T cell culture. All cell culture was performed at 37 °C in a humidified atmosphere containing 5% CO_2_. HEK293T cells were grown in DMEM supplemented with 10% fetal bovine serum (FBS), 10 mM L-glutamine, and 1% penicillin–streptomycin. Cells were cultured at a density of 2 × 10^5^–1 × 10^6^ cells/mL. Transfection was carried out in DMEM without L-glutamine and antibiotics.

Plasmid cloning and viruses. The plasmid of LifeAct-mCherry was a kind gift of Prof. Stephan Huveneers [43] and was subcloned into the lentiviral destination vector (Addgene, Cat# 17454) using gateway cloning. shRNA constructs for BRAF were designed by using the cDNA sequence of human BRAF on the web page http://splashrna.mskcc.org/ [44] (date accessed: 8 June 2019) and cloned into the SGEP (miR-E LentipRRL) vector (kind gift of Prof. Manuela Baccarini) based on Fellmann et al. [45]. Briefly, the amplified shRNAs were cloned into the SGEP vector by using EcoRI and XhoI with the NEB Builder HiFi DNA Assembly protocol of New England Biolabs.

Lentiviral supernatants were generated by co-transfection of HEK293T cells with a three-vector lentiviral system: using the specific expression vector combined with the lentiviral packaging and envelope plasmids pRSV-Rev, pMDLg/pRRE and pCMV-VSV-G (kind gift of Prof. Guillaume Charras).

Lentiviral Packaging. Lentiviral packaging of all constructs was performed using HEK293T cells. Transfection was carried out using PEI as a transfection reagent, which was prepared according to [46]. Then, 2.7 × 10^6^ HEK cells were seeded in a T25 flask the day before transfection. The plasmid mixture contained 4 µg construct plasmid, 1.5 µg VSV-G, 0.75 µg Rev and 0.75 µg RRE virus plasmids. PEI was used in 1:2 ratio. HEK transfection medium was exchanged for DMEM medium containing 10% FBS 3–4 h after transfection. After 24 h, HUVEC medium was applied on the transfected HEK cells. Viral supernatant was harvested after a total of 48 h and filtered through a 0.45 µm filter. For transduction of HUVECs, cells were cultured in 50% HUVEC medium and 50% viral supernatant (shRNA virus: Lifeact = 1:2) for 24 h, supplemented with polybrene (Santa Cruz Biotechnology, Cat# sc-134220, 4 µg/mL final concentration). For transduction of HUVECs, the virus was mixed with trypsinized HUVECs and 2 × 10^5^ cells were seeded on a 6-well plate.

siRNA transfection. siRNA transfection was carried out in OPTI-MEM (ThermoFisher Scientific, Cat# 31985062 Tkoyo Japan) by using Lipofectamine RNAiMAX (ThermoFisher Scientific, Cat# 13778030 Tkoyo Japan) and siRNA (25 nM). Cells were incubated with the siRNA–RNAiMAX mixture for 4 h and seeded on the cytostretcher chambers with the cell numbers provided above.

Monolayer stretching. HUVEC monolayers were cultured in special chambers used in the Cytostretcher LV instrument of CuriBio (former Nanosurface Biomedical Inc. Seattle, WA, USA). The bottom of the chamber was treated with 0.2 mg/mL polydopamine solution (dissolved in 10 mM TRIS buffer, pH 8.5) for 2.5 h and washed 3 times with sterile water to create a hydrophilic surface for gelatin coating. Stretching was carried out with 0.5%/s velocity and the monolayer was kept stretched for different durations (0–30 min) before cell lysis happened. For Western blot analysis, 12 mm × 12 mm chambers were used (4.5 × 10^5^ cells). Monolayers with genetically encoded proteins (i.e., LifeAct) were monitored after stretching with a STED superresolution microscope (Expertline, Abberior Instruments, Göttingen Germany) in 5 mm × 5 mm chambers (4.35 × 10^4^ cells). ROCK inhibitor treatment was carried out for 1 h prior to stretching with 10 µM Y27632.

Live imaging. Live imaging of stretching was performed on a Nikon Ti2 confocal microscope. The field of view for imaging was a 210 µm × 210 µm area (resolution: 1050 × 1050 pixels) and pictures were taken every 2 min by using a 20× lens (numerical aperture: 0.75). The monolayer was imaged for a total of 10 min before and for 30 min after stretch. The monolayer was refocused after stretching, which delayed the recording by 2 min after stretch. Based on quantification of the fluorescent data, live imaging did not decrease the total fluorescence intensity below 95% of the value obtained at the beginning of the recording.

Immunofluorescence staining of fixed monolayers. Cells grown in cytostretcher chambers (unstretched or stretched) were fixed with Image-iT™ Fixative Solution (ThermoFisher Scientific, Cat# R37814 Tokyo Japan) for 15 min. After that, cells were washed with HBSS, permeabilized (0.25% Triton X-100 in TBS-T, 10 min RT), blocked (1% BSA in TBS-T, 1 h RT) and incubated with the primary antibodies (dilutions prepared in 1% BSA-TBS-T for VE-cadherin—1:400, pMLC—1:200, PECAM-1—1:2000, p-cofilin—1:100, cofilin—1:200; incubation was carried out overnight at 4 °C). After thorough washing in TBST, cells were stained simultaneously with the appropriate secondary antibodies (dilutions were prepared as 1:2000) and phalloidin (1:1000 in 1% BSA-TBST) for 1 h at RT and washed in TBS-T and PBS prior to imaging.

Immunoblotting. Cells were harvested in 25 mM HEPES, pH 7.4, 150 mM NaCl, 1 mM EGTA, 1% NP-40, 10% glycerol, supplemented with the following protease and phosphatase inhibitors: 10 mM sodium pyrophosphate, 10 mM sodium fluoride, 5 mM sodium vanadate, 1 mM PMSF and cOmplete, EDTA-free protease inhibitor cocktail (Sigma, Tokyo, Japan). Lysates were centrifuged with 5000× *g* for 5 min at 4 °C and the supernatant was snap frozen for further immunoblotting.

Proteins were separated using standard SDS-PAGE gel electrophoresis with 12% SDS-PAGE gels, transferred to PVDF membranes for immunoblot analysis using a wet blot transfer system (BioRad, Hercules, CA, USA) and stained with specific primary antibodies as indicated in each figure. Afterwards, membranes were incubated with the appropriate HRP-conjugated secondary antibodies. Bands were visualized by using a chemiluminescence substrate and developed on Hyperfilms. Bands were quantified using ImageJ.

### 4.3. Quantifications

Analysis of junction length, actin fluorescence intensities and gap size. Lengths of the junctions were determined according to the plasma membrane staining (DeepRed Cellmask Tokyo Japan) and they were analyzed together with the actin fluorescence intensities by using Fiji. For quantification of fluorescence intensities, only those junctions were selected for analysis where the junction was parallel (horizontal) or perpendicular (vertical) to the stretch direction or it was tilted before stretch and became parallel or perpendicular upon stretch. Therefore, only those cells were considered in the quantifications where the junctions were clearly parallel or perpendicular before or after stretch. Junctional actin fluorescence intensities were corrected for photobleaching, measured as a fluorescence intensity change for the whole cell during the time course of stretch.

Gaps were identified by using the pictures of plasma membrane staining of selected time points of the time-lapse images. After adjusting the brightness, the threshold was set to convert the image to a binary one. As a result, the gap area appears as black, and was quantified in Fiji using the Particle Analysis plugin and then normalized to total junction lengths. Only junctions between EGFP-expressing cells were considered. All gap areas under 200 µm^2^ were used for calculation.

Data are presented in all figures as mean ± SEM for biological replicates. Statistical analyses were carried out in GraphPad Prism 4 (version 4.01). Significance was determined by a two-tailed *t*-test in Microsoft Excel. Differences between groups were considered statistically significant if *p* < 0.05.

## Supplementary Materials

The following are available online at https://www.mdpi.com/article/10.3390/ijms22168989/s1.
