# Supplementary material for: BRAF Modulates Stretch-Induced Intercellular Gap Formation through Localized Actin Reorganization
Source: Int J Mol Sci. 2021 Aug 20;22(16):8989. doi: 10.3390/ijms22168989 (PMC8396467; doi:10.3390/ijms22168989)
Supplement: Supplementary file 1 [file ijms-22-08989-s001.zip › Supplementary material.pdf]

Supplementary material

BRAF Modulates Stretch-Induced Intercellular Gap Formation through Localized Actin Reorganization

Anna Hollósi, Katalin Pászty, Miklós Kellermayer, Guillaume Charras and Andrea Varga

Figure S1: related to Figure 3 Visualization and quantification of actin reorganization in shControl and shBRAF HUVECs

Figure S2: related to Figure 4 Effect of stretch on the localization of p-cofilin and actin in control (siscr) and siBRAF monolayers

Videos S1 and S2: Time-lapse images of shControl (Video S1) and shBRAF (Video S2) monolayers labeled with LifeAct-mCherry illustrating actin localization before and after stretch

Videos S3 and S4: Time-lapse images of shControl (Video S3) and shBRAF (Video S4) monolayers labeled with DeepRed CellMask (gray) and shRNA (green) illustrating intercellular gap formation

Figure S1: related to Figure 3 Visualization and quantification of actin reorganization in shControl and shBRAF HUVECs

(A) Western blot analysis showing the efficiency of BRAF knockdown in HUVECs infected with control and 2 different shRNAs for BRAF. (B) and (C) are the zoomed-out views of Figure 3A and B. Monolayer (plasma membrane colored as gray) expressing shControl (B) or shBRAF (C) RNAs (green) and LifeAct-mCherry (red) is shown before stretch. LifeAct-mCherry is shown after stretch for the indicated time points. Scale bar is 50 μm. (D) and (E) show quantified actin fluorescence intensities in the stretched (horizontal) junctions in shControl (D) and shBRAF (E) cells, respectively. (F) and (G) show quantified actin fluorescence intensities in the unstretched (vertical) junctions in shControl (F) and shBRAF (G) cells, respectively.

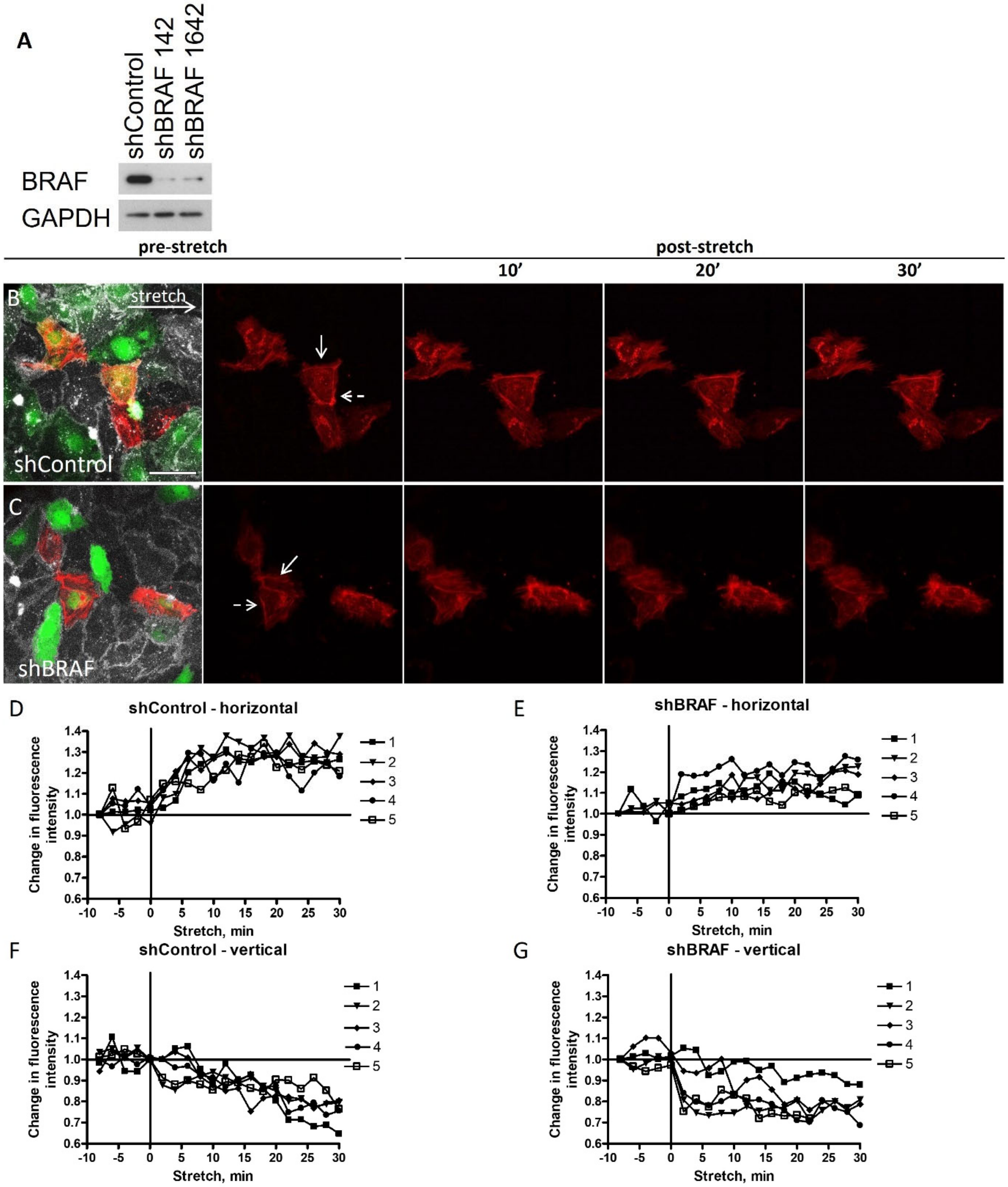

Figure S2: related to Figure 4 Effect of stretch on the localization of p-cofilin and actin in control (siscr) and siBRAF monolayers

Immunofluorescence images of unstretched and stretched siScr and siBRAF monolayers stained for p-cofilin. (B) Merged immunofluorescence images of VE-Cadherin (blue) and actin (green) of unstretched and stretched siScr and siBRAF monolayers.

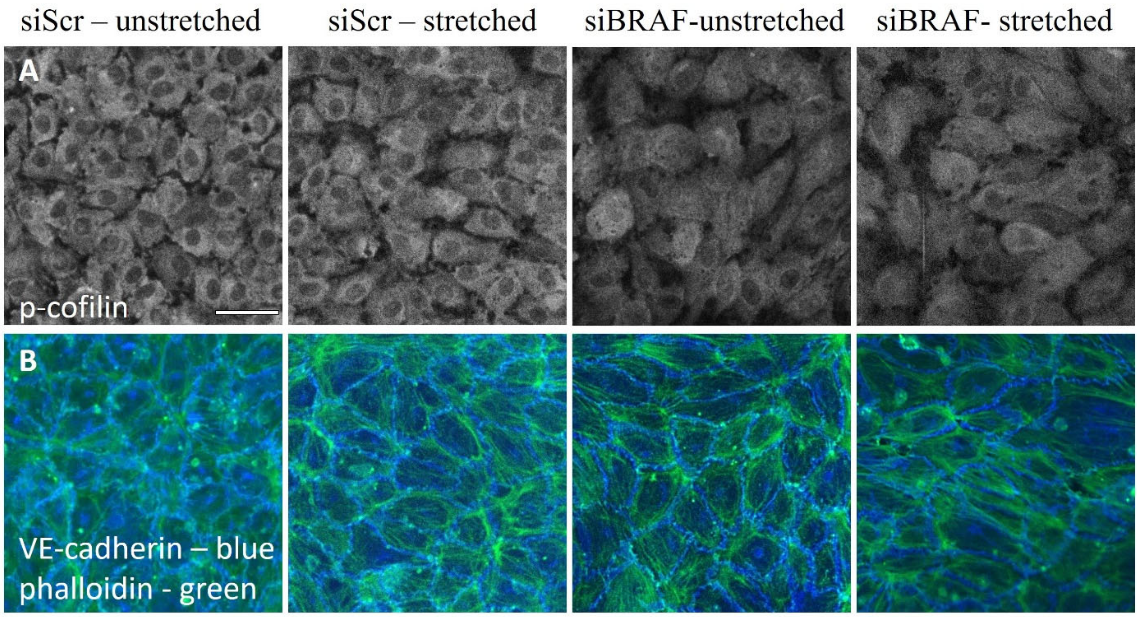

Videos S1 and S2: Time-lapse images of shControl (Video S1) and shBRAF (Video S2) monolayers labeled with LifeAct-mCherry illustrating actin localization before and after stretch

Videos S1 and S2 show the time-lapse images of actin fluorescence (LifeAct-mCherry) of shControl and shBRAF monolayers taken every 2 minutes (stretch was initiated at time zero and the first picture was recorded 2 minutes after stretch), respectively. Specific time points of these videos are shown in Fig S1B, and C. Zoomed-in views can be seen in Fig 3A and B.

Videos S3 and S4: Time-lapse images of shControl (Video S3) and shBRAF (Video S4) monolayers labeled with DeepRed CellMask (gray) and shRNA (green) illustrating intercellular gap formation

Videos S3 and S4 show the time-lapse images of shControl and shBRAF monolayers taken every 2 minutes (stretch was initiated at time zero and the first picture was rec-orded 2 minutes after stretch), respectively. Plasma membrane stain (DeepRed CellMask staining) is colored as gray, while shRNA is labeled by green (EGFP is co-expressed with the shRNAs).
